# A stacking based deep learning framework integrating random search neural architecture search for meniscus tear diagnosis

**DOI:** 10.1002/acm2.70711

**Published:** 2026-07-22

**Authors:** Ebubekir Seyyarer, Hasan Genç, Faruk Ayata

**Affiliations:** ^1^ Department of Computer Engineering Van Yüzüncü Yıl University Van Türkiye; ^2^ Radiology Clinic Elazığ Fethi Sekin City Hospital Elazığ Türkiye; ^3^ Department of Computer Technologies Van Yüzüncü Yıl University Van Türkiye

**Keywords:** deep learning, elasticnet meta‐learner, explainable AI, knee MRI, late fusion, meniscus tear diagnosis, neural architecture search, stacking ensemble

## Abstract

**Background:**

Accurate and rapid diagnosis of meniscal tears is crucial for effective management of sports‐related injuries and degenerative knee disorders. Magnetic resonance imaging (MRI) is widely used for meniscus evaluation; however, manual interpretation is time‐consuming and subject to inter‐observer variability. Automated and reliable classification systems may therefore support clinical decision‐making.

**Purpose:**

This study aims to develop a robust and interpretable deep learning framework for the automatic four‐class classification of meniscal conditions using MRI images.

**Methods:**

A total of 2,000 knee MRI images obtained from a tertiary care university hospital were categorized into four classes: Grade I, Grade II, Grade III, and normal meniscus. A Random Search‐based Neural Architecture Search (RS‐NAS) strategy was used to generate task‐specific CNN architectures. The class probability outputs of the top‐performing NAS‐derived CNN models were combined using a late‐fusion stacking strategy, with ElasticNet employed as the meta‐learner. The proposed framework was evaluated using both a single‐run assessment and a repeated validation protocol consisting of 10 independent runs with 5‐fold cross‐validation. Performance was assessed using ACC, F1‐score, MCC, AUC, and PR‐AUC. Statistical comparisons and explainability analyses, including Grad‐CAM and ElasticNet feature importance, were also performed.

**Results:**

In the single‐run evaluation, the ElasticNet‐based stacking model achieved an ACC of 0.9300, F1‐score of 0.9304, and AUC of 0.9913. Under the repeated 10‐run 5‐fold cross‐validation protocol, the proposed model obtained an ACC of 0.9083 ± 0.0019, F1‐score of 0.9084 ± 0.0018, MCC of 0.8783 ± 0.0024, AUC of 0.9883 ± 0.0008, and PR‐AUC of 0.9719 ± 0.0020. The proposed stacking framework outperformed the best NAS CNN, DenseNet121‐TL, averaging ensemble, and majority voting ensemble across the main evaluation metrics. Grad‐CAM visualizations and feature importance analysis further indicated that the model relied on clinically meaningful image regions and informative base‐model predictions.

**Conclusions:**

The integration of RS‐NAS with ElasticNet‐based late‐fusion stacking provides a stable, high‐performing, and interpretable framework for four‐class meniscus MRI classification. Although the single‐run evaluation showed higher peak performance, the repeated cross‐validation results provide a more reliable estimate of model robustness and generalization. Future studies should validate the proposed framework using independent multi‐center datasets and different imaging protocols.

## 1 INTRODUCTION

Meniscus tears are one of the most common soft tissue injuries to the knee. They usually occur as a result of sports activities or sudden rotational movements, or as part of the degenerative processes associated with ageing. If left untreated, they can lead to serious consequences such as permanent pain, joint stiffness and impaired function. Therefore, an early and accurate diagnosis is critical for successful treatment and for improving the patient's quality of life. The complex structure of meniscus tissue and the varying severity of injuries make clinical evaluation difficult. In this context, magnetic resonance imaging (MRI) is the most reliable and widely used method for analysing soft tissues. MRI allows high‐resolution imaging of the meniscus's anatomical integrity and morphological changes. However, accurate interpretation of these images largely depends on the radiologist's experience, which can lead to diagnostic errors and wasted time.

In recent years, rapid advancements in artificial intelligence (AI) technologies have ushered in a new era for medical imaging.^1^ In particular, deep learning (DL) models can automatically analyse complex MRI data,^2^, ^3^ recognising multidimensional patterns in images and significantly improving diagnostic processes. DL algorithms achieve high levels of accuracy in detecting meniscus lesions, reducing the need for manual assessment and speeding up the clinical decision‐making process. Thus, deep learning‐based systems offer significant innovations in terms of both diagnostic accuracy and healthcare efficiency, standing out as a modern approach to identifying and classifying meniscus tears. As AI‐based methods for the early diagnosis of meniscus tears become more prevalent, a variety of deep learning architectures have been developed in this field. Examining these studies reveals that various convolutional neural network (CNN) derivatives, vision transformer models, automated feature extraction approaches and hybrid methods have been successfully applied to classify meniscus pathologies. This is mainly because the complex anatomical structure of the meniscus involves multidimensional image characteristics that are difficult to extract manually, so there is an increasing need for automated methods. Furthermore, since the quality of diagnostic MRI images can vary depending on variables such as imaging protocol, patient characteristics and device parameters, the generalisability of the models is of great importance. In this context, the main contributions of our study can be summarised as follows:

✓ A four‐class meniscus MRI classification framework was developed using a clinical dataset consisting of Grade I, Grade II, Grade III, and normal meniscus images validated by expert radiologists.
✓ A Random Search‐based Neural Architecture Search strategy was employed to automatically generate task‐specific CNN architectures and reduce manual trial‐and‐error during model design.
✓ The five best NAS‐derived CNN models were integrated using a late‐fusion stacking strategy, where their class probability outputs were combined and processed by an ElasticNet meta‐learner.
✓ The proposed framework was evaluated using both single‐run assessment and a repeated validation protocol consisting of 10 independent runs with 5‐fold cross‐validation, providing a more robust estimate of model stability and generalization.
✓ Comprehensive performance evaluation was conducted using ACC, precision, recall, F1‐score, MCC, AUC, and PR‐AUC, together with statistical comparisons between models.
✓ An ablation‐style analysis was performed to assess the individual contribution of NAS, simple ensemble strategies, and the final stacking framework.
✓ Explainability analyses were incorporated using Grad‐CAM for CNN‐based predictions and feature importance analysis for the ElasticNet meta‐model, improving the clinical interpretability of the proposed framework.
✓ Representative correctly classified and misclassified MRI examples were provided to visually assess model behavior and identify challenging borderline cases.
✓ The proposed approach demonstrated improved stability and predictive performance compared with individual CNN models, transfer learning baselines, and conventional ensemble strategies.

The remainder of the study is structured as follows: Section 2 presents a review of deep learning‐based approaches for meniscus tear detection and related medical image classification studies. Section 3 describes the MRI dataset, preprocessing steps, Random Search‐based NAS procedure, transfer learning baselines, late‐fusion stacking strategy, evaluation protocol, and performance metrics. Section 4 presents the experimental results, including single‐run and repeated 5‐fold cross‐validation evaluations, statistical comparisons, ablation analysis, error analysis, and explainability results. Finally, Section 5 discusses the main findings, limitations, clinical relevance, and future research directions.

## 2 LITERATURE REVIEW

Artificial intelligence (AI) and deep learning (DL) technologies are driving revolutionary change in the field of medicine. Numerous academic studies have examined the effectiveness of these technologies, particularly in diagnosis, imaging and clinical decision support systems.^4^ Several studies have demonstrated that AI serves as a supportive tool, enhancing the accuracy of physicians in interpreting images and reducing radiological error rates. Similarly, a study examining the ethical, reliable and transparent aspects of AI revealed that explainable artificial intelligence (XAI) is crucial for the adoption of healthcare services.^5^

A bibliometric analysis evaluating the applications of AI in healthcare over time revealed a significant increase in publications in areas such as clinical decision support systems, patient monitoring and image classification over the past five years.^6^ Concurrently, a systematic study examining the integration of different imaging techniques (CT, MRI and x‐ray) with AI revealed the impact of multimodal learning, transfer learning and attention mechanisms on performance.^7^

Deep learning methods, particularly convolutional neural networks (CNNs) and, more recently, vision transformers (ViTs), have attracted attention due to their ability to perform classification and segmentation tasks on medical images with high accuracy. Numerous studies have demonstrated the success of DL methods in many subfields, particularly in radiology.^8^ Significant improvements in the early diagnosis of cancer have been achieved through the use of 3D CNNs and transfer learning methods.^9^ These developments demonstrate the adaptability of DL to specific clinical areas, particularly orthopaedic imaging. Meniscus tears are one of the most common knee joint pathologies, and MRI images are frequently used in their diagnosis. As interpretations can vary among radiologists when using traditional methods, DL models have the potential to provide more objective and reproducible solutions. In this context, a multitasking deep learning model developed by Tack et al.,^10^ detected medial and lateral meniscus tears with high accuracy according to their anatomical location. Similarly, the Mask R‐CNN‐based model developed by Li et al.^11^ achieved a classification accuracy of over 85% in distinguishing between tears and degenerative changes to the meniscus. More recently, Güngör et al. (2023) in the study achieved high accuracy in meniscus tear detection using advanced deep learning models, demonstrating reliable performance even with a relatively small dataset^12^.

Although these studies offer promising results for diagnosing meniscus tears, many models rely solely on image data, ignore patient‐specific variability and are limited in their generalisability, which are all significant constraints. Furthermore, the explainability and clinical integration processes are not yet sufficiently advanced. For these reasons, more holistic approaches are needed in terms of both model architecture and data diversity.

Table 1 compares fundamental studies on the classification of meniscus tears from MRI images in the literature with the proposed RS‐based NAS + Stacking model. The comparison shows that, while classical CNN and transfer learning approaches achieved accuracy rates of 86%–89%, The proposed hybrid model achieved 90.83% ± 0.19% ACC, 98.83% ± 0.08% AUC, and 90.84% ± 0.18% F1‐score under the repeated 10‐run 5‐fold cross‐validation protocol, while the single‐run evaluation reached 93.75% ACC and 0.9909 AUC.

**TABLE 1:** Comparison of MRI‐based meniscus studies and the proposed method.

Autors / Year	Method / Models	Dataset	ACC	AUC / F1
Li et al.^11^	Mask R‐CNN	MRI (Ortho. Surg. Research)	89.2	0.93 / 0.90
Ismail et al.^13^	VGG‐16 (Transfer Learning)	Clinical MRI dataset	86.5	0.91 / 0.88
Fritz et al.^14^	2D CNN (Fully Automated)	100 knee MRIs + arthroscopy validation	86.0	0.88 / 0.86
Li et al.^11^	3D Mask‐RCNN (Segmentasyon + Tespit)	546 MRIs + surgical confirmation	89.5	0.94 / 0.91
This Study (2025)	RS‐based NAS + Stacking (ElasticNet)	2000 MRIs (proprietary dataset)	90.83 ± 0.19	98.83 ± 0.08 / 90.84 ± 0.18

*Note*: The values for this study represent the repeated validation results obtained from 10 independent runs with 5‐fold cross‐validation. In the single‐run evaluation, the proposed model achieved ACC = 93.00%, AUC = 0.9913, and F1‐score = 0.9304.

To ensure fairer comparability, Table 1 was restricted to MRI‐based meniscus studies. General NAS, stacking, and non‐meniscus datasets are discussed in the literature review as broader methodological background rather than included in the direct comparative table.

## 3 MATERIALS AND PROPOSED METHODOLOGY

**FIGURE 1:**
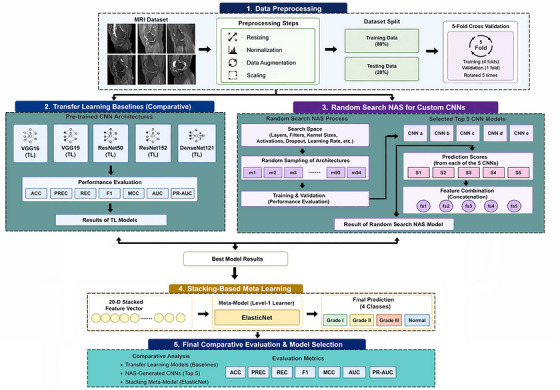
Overall experimental workflow of the proposed RS‐based NAS and stacking framework, including data preprocessing, 5‐fold cross‐validation, transfer learning baselines, NAS‐based CNN generation, and final model comparison.

This section presents the methodological framework of the proposed study in detail. It includes the MRI dataset, preprocessing operations, transfer learning based baseline evaluation, the Random Search based NAS procedure for generating custom CNN architectures, the stacking based meta‐learning stage, and the final comparative model selection process. As shown in Figure 1, the overall workflow starts with dataset preparation, preprocessing, dataset splitting, and 5‐fold cross‐validation, proceeds through the parallel evaluation of transfer learning and NAS based CNN models, and ends with ElasticNet based stacking and comparative assessment using the selected evaluation metrics.

Figure 1 shows the general framework of the proposed study, which consists of five main stages ranging from data preprocessing to final model evaluation. In the first stage, MRI images undergo preprocessing steps such as resizing, normalisation, data augmentation and scaling. The dataset is then divided into training and test subsets and 5‐fold cross‐validation is applied to provide a more reliable evaluation of the model's performance. This approach aims to analyse the model's performance under different data divisions and reduce the risk of overfitting.

The second stage involves creating transfer learning‐based baseline models for comparison purposes. In this context, commonly used pre‐trained deep learning architectures, such as VGG, ResNet and DenseNet, are employed to fine‐tune the models. The models are then evaluated using various performance metrics, including accuracy (ACC), precision (PREC), sensitivity (REC), the F1 score, the Matthews correlation coefficient (MCC), the area under the curve (AUC) and the PR‐AUC. This stage provides a strong baseline for subsequent analyses.

The third stage involves designing custom CNN architectures using a Random Search‐based Neural Architecture Search (NAS) method. A search space is defined that includes hyperparameters such as layer structures, filter sizes, activation functions and learning rates. Numerous candidate models are then generated from this space by random sampling. These models are then trained and evaluated, and those showing the best performance are selected. The prediction outputs of these selected CNN models are then combined for use in the next stage.

In the fourth stage, a stacking‐based meta‐learning approach is employed. The probability outputs from the selected CNN models are combined to create a feature vector, which is then used as input for the ElasticNet meta‐model. This meta‐model then learns complementary information from the base models to produce the final classification output for the four classes.

In the final stage, a comprehensive comparative evaluation is conducted. The proposed stacking approach is compared with transfer learning‐based models and CNN models obtained with NAS using the same evaluation metrics. This stage ensures a fair and consistent comparison of all models, providing systematic evidence of the effectiveness of the proposed method.

**FIGURE 2:**
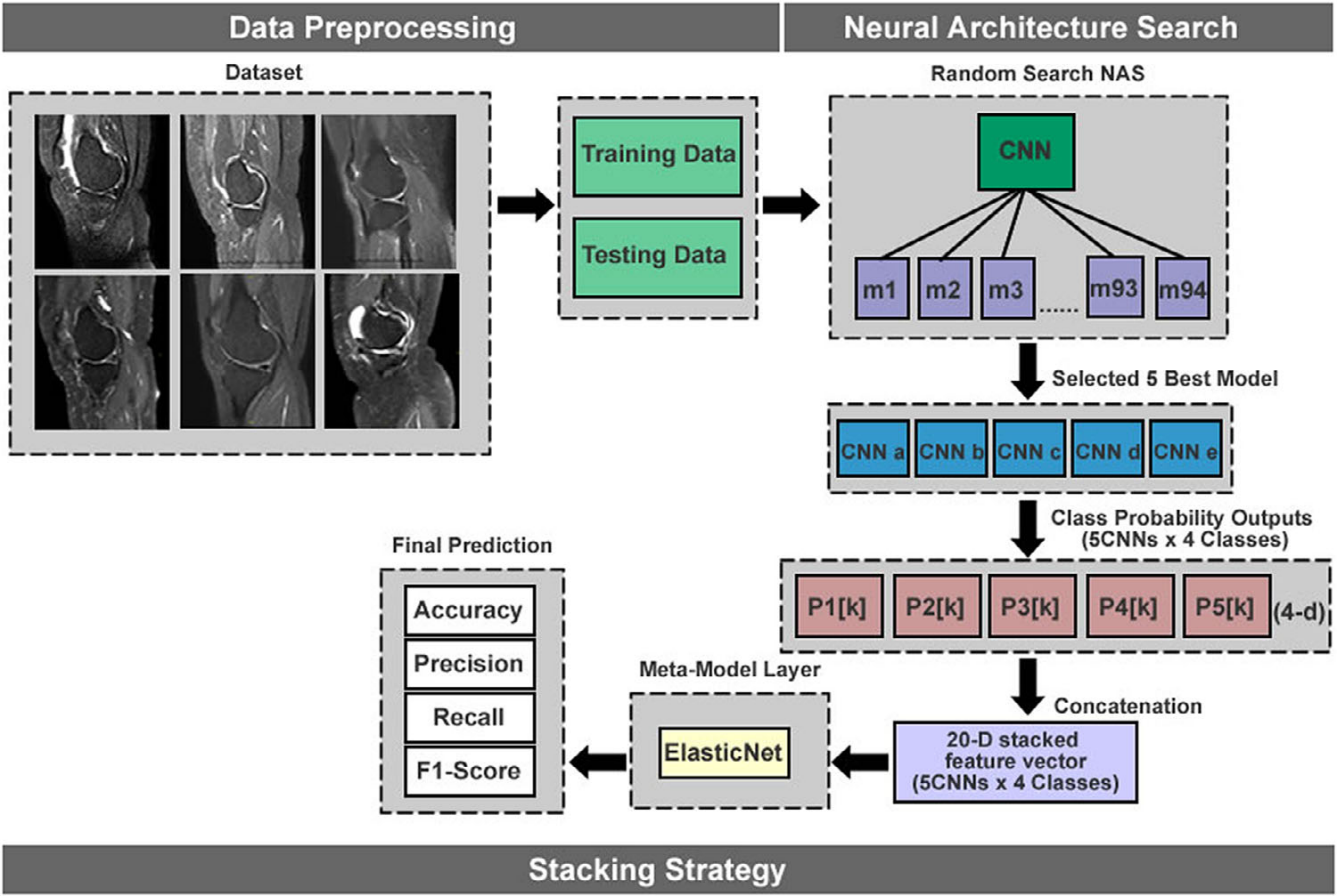
Stacking‐based late fusion architecture. The class probability outputs produced by the five CNN models are concatenated to form a 20‐dimensional stacked feature vector (5 models × 4 classes), which is used as input to the ElasticNet meta‐learner.

The workflow shown in Figure 2 illustrates the general workflow of the proposed multi‐stage deep learning framework for meniscus MRI classification. The modelling process consists of three main sections: data preprocessing, Neural Architecture Search (NAS), and a stacking‐based meta‐classification strategy.

In the first stage, the meniscus MRI dataset is prepared and divided into training and testing subsets. The images are then preprocessed to make them compatible with the model input, with operations such as resizing, normalization, data augmentation, and scaling being applied. This process provides a more homogeneous and reliable data structure for model training and evaluation.

In the second stage, several different CNN architectures (m1–m94) are generated using a Random Search‐based NAS procedure. From these candidate architectures, the five best‐performing CNN models (CNN a–e) are selected according to their validation performance. These selected models are then used as the base learners in the subsequent stacking phase.

In the third stage, the class probability outputs produced by the five selected CNN models are combined through concatenation. Since each CNN generates a four‐class probability output, the concatenation of five models results in a 20‐dimensional stacked feature vector for each sample (5 models × 4 classes). These stacked features are then passed to the meta‐model layer, where ElasticNet is used as the meta‐model. The meta‐model processes the combined representation to produce the final prediction. The overall system performance is then evaluated using key classification metrics such as accuracy, precision, recall, and F1‐score.

### 3.1 Dataset

An accurate diagnosis of meniscus tears can be achieved using MRI. This non‐invasive, radiation‐free imaging technique shows soft tissues with high clarity, allowing for a detailed examination of meniscus damage.^15^, ^16^, ^17^ Different MRI protocols enable evaluation of characteristics such as tear type and extent, and connection to surrounding structures. However, as the interpretation of the images requires expertise, the process can be lengthy and vary depending on the evaluator.

**FIGURE 3:**
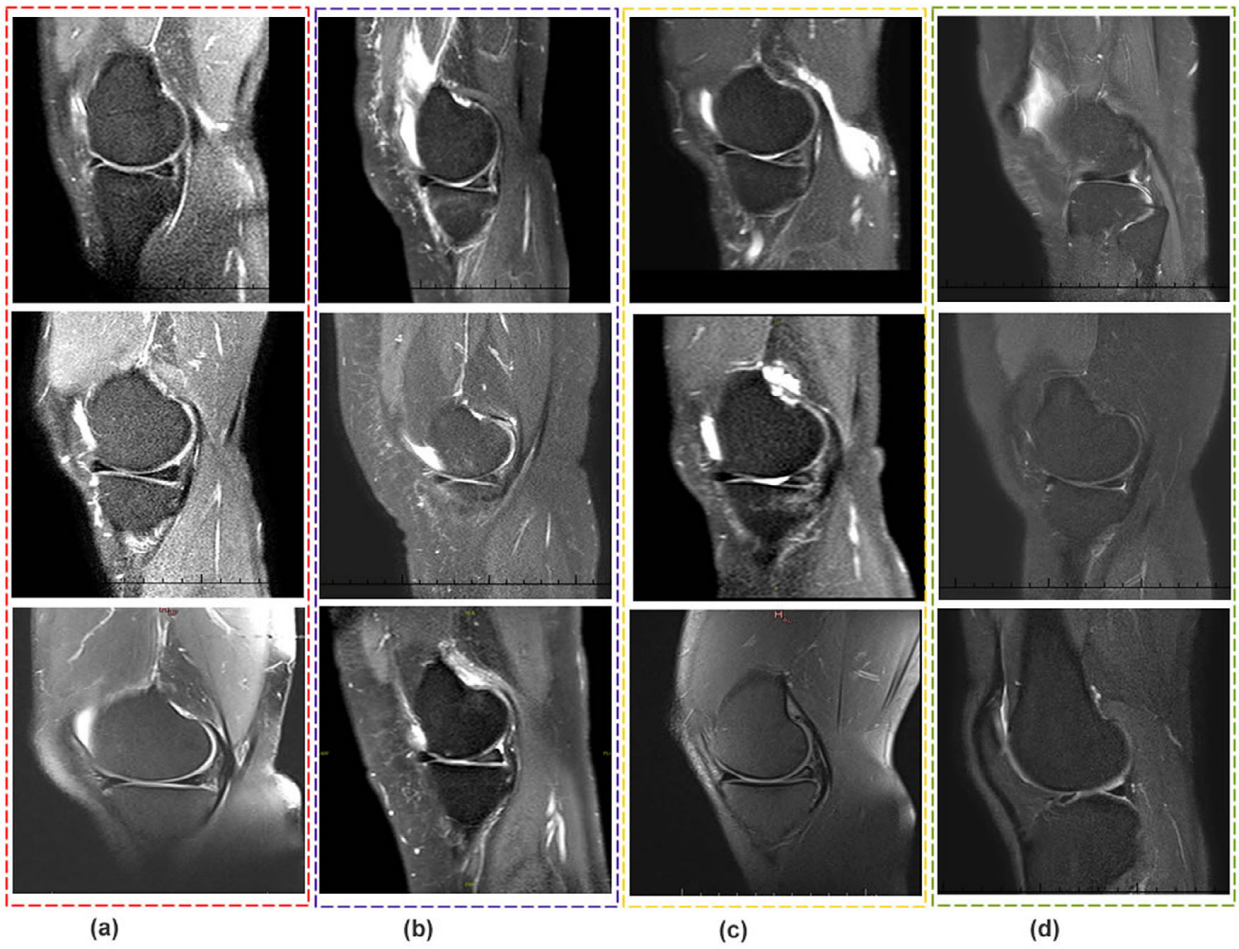
(a) I. Grade, (b) II. Grade, (c) III. Grade, (d) Normal class meniscus dataset.

This study used a dataset comprising 2,000 meniscus MRI images obtained at Fırat University Hospital. The images were categorized into four groups for the classification of meniscus tears: Grade I, Grade II, Grade III, and normal (see Figure 3). Experienced radiologists excluded images of low diagnostic value or those containing artifacts. Only scans with clearly visible tears and sufficient image quality were included, ensuring a reliable basis for model development.

All MRI images were resized to 224 × 224 pixels and normalized to the range [0, 1] prior to model training. Data augmentation was applied only to the training subset in order to improve generalization and reduce the effect of class imbalance, while no augmentation was applied to the validation or test subsets. The augmentation pipeline included random rotations (±15°), horizontal flipping, zooming of up to 10%, and brightness adjustments. Before augmentation, the class distribution was imbalanced, with fewer samples in the higher‐grade tear categories. To reduce this imbalance, additional augmented samples were generated only for the underrepresented classes until a more balanced training distribution was achieved. Accordingly, the class balancing procedure was restricted to the training data and did not affect the validation or test sets.

To ensure robust performance evaluation, a repeated 5‐fold cross‐validation strategy was adopted. In each fold, the dataset was divided into training and test subsets, where four folds were used for training and one fold was kept as an independent test set for final evaluation. Within the training portion, 20% of the data was further separated as a validation set to support early stopping, hyperparameter tuning, and model selection. The validation set was used only during training and was not involved in the final performance evaluation. The test fold was kept completely unseen during the NAS and stacking stages and was used exclusively for final evaluation. This strict separation ensured that no data leakage occurred between training, validation, and test phases.

To minimize leakage risk, the dataset was constructed such that each patient contributed only a single MRI image. Therefore, no patient appeared more than once in the dataset, and repeated images from the same individual were not present across the training, validation, and test subsets. In addition, the test portion of each fold was kept completely held out and was not used during NAS, model selection, or stacking training. During the NAS phase, only the training and validation subsets were used for architecture search and performance evaluation. Similarly, the stacking model was trained using predictions generated from the training data without accessing the test data. The test data were used exclusively for final performance evaluation within the corresponding fold.

Due to data sharing restrictions associated with the clinical MRI dataset, the full source code and data repository are not publicly available. However, all implementation details required to reproduce the experimental workflow have been described in the manuscript, and the code can be made available upon reasonable request.

### 3.2 Neural architecture search

Although Neural Architecture Search (NAS) is effective in identifying high‐performing CNN architectures by exploring a large hyperparameter space, it typically focuses on selecting a single best‐performing model. However, relying on a single model may limit generalization, especially in medical imaging tasks where data variability is high.

To address this limitation, ensemble learning techniques such as stacking can be employed. Stacking combines the outputs of multiple base models and leverages a meta‐learner to capture complementary patterns among them. In this study, Random Search‐based NAS is used to generate diverse and high‐performing CNN architectures, while stacking is utilized to integrate their predictions and improve overall robustness and generalization performance.

Thus, NAS and stacking play complementary roles: NAS enhances model discovery, whereas stacking enhances model integration.

NAS is a method that enables the automated generation of CNN structures. It has surpassed human‐designed models in many areas, such as object detection,^18^ image classification^19^, ^20^ and semantic parsing.^19^ The RS algorithm employed in this study is a random sampling‐based method for optimising hyperparameters that gained importance following the findings presented by Bergstra and Bengio in 2012. Unlike grid search, which systematically scans points in the parameter space, this method can more quickly reach critical hyperparameters affecting model performance by randomly selecting from predetermined intervals. In high‐dimensional search domains in particular, it achieves more efficient exploration while reducing computational costs by taking advantage of the fact that only a small subset of parameters is decisive in model success. Due to its ease of implementation, suitability for parallelisation and wide range of applications, from deep learning to NAS processes, RS is currently regarded as a straightforward yet effective initial optimisation method.^21^

**FIGURE 4:**
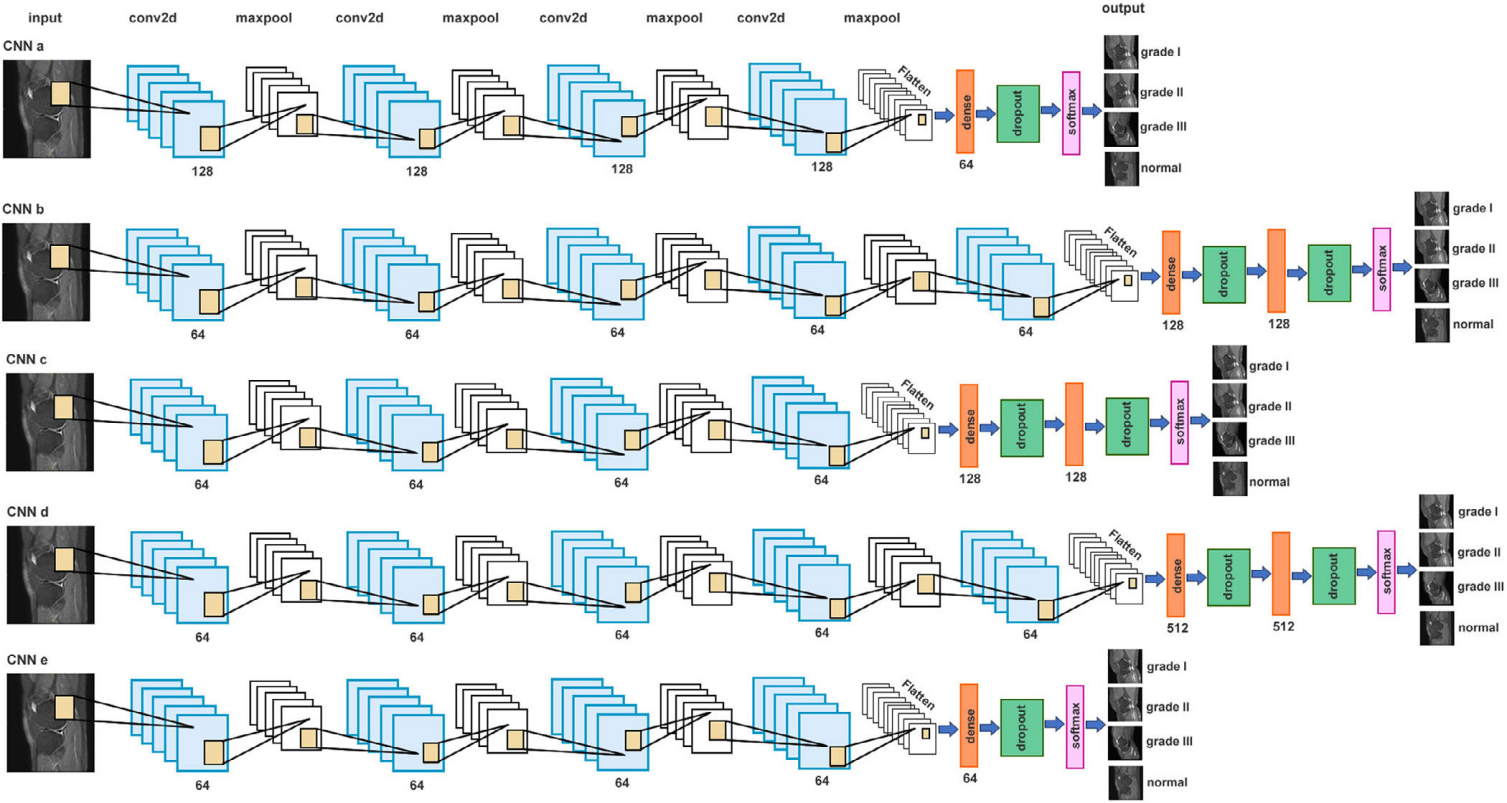
Layer structure of the five best selected models.

Figure 4 shows the detailed layer structures of the five CNN architectures, while Table 2 summarizes their key architectural differences. Each architecture uses meniscus MRI images as input and produces a multi‐class output (grade I, grade II, grade III and normal). The architectures consist of convolutional (Conv2D) and maximum pooling (MaxPool) layers, fully connected (dense) layers, dropout mechanisms and a softmax classifier in the final stage.

**TABLE 2:** Comparative summary of the architectural configurations of the five CNN models (CNN a to CNN e), including the number of convolutional blocks, filter sizes, dense layers, and dropout settings.

Model	Conv Blocks	Filters	Dense Layers	Units	Dropout
CNN a	4	128	1	64	0.5
CNN b	5	64	2	128	0.5
CNN c	4	64	2	128	0.5
CNN d	5	64	2	512	0.5
CNN e	4	64	1	64	0.5

Architecture a has a structure consisting of four sets of Conv2D and MaxPool blocks, with 128 filters used in each block. This high number of filters, particularly in the convolutional layers, enables the model to perform richer feature extraction. Following the flattening process, the model transitions to a classification layer comprising two fully connected layers and a dropout layer to mitigate overfitting.

CNN b consists of 19 layers and uses 64 filters in each convolutional block. This structure is lighter than that of CNN A and contains only a single fully connected layer. Its convolution pattern comprises five Conv2D + MaxPool blocks, with a dropout applied before the softmax classifier.

CNNc is a more compact CNN structure, comprising a total of 16 layers. It comprises four convolution blocks, followed by a single dense layer. The number of filters is fixed at 64. Due to its lightweight structure, it is more computationally efficient.

CNN d has a similar filter arrangement to CNN b, but differs in that it has a deeper fully connected layer structure. Two dense layers (with 128 neurons) and subsequent dropout layers enable the model to learn more abstract representations. This increases its discriminatory capacity, particularly when there are subtle differences between classes.

CNn e is the deepest and largest of the five CNN architectures. It contains three fully connected layers with 512 neurons following convolution blocks and therefore has the highest number of parameters. Its multi‐layered dense structure offers stronger representational capacity for capturing complex patterns.

All CNN models generate probability distributions belonging to four classes with softmax activation in the final stage. The main differences between the architectures manifest in structural components such as the number of layers, filters and fully connected layers, as well as the intensity of dropout usage. These variations enable optimisation by the NAS process, ensuring diversity and generating complementary information in the stacking strategy.

It should be noted that the CNN architectures generated through the Random Search based NAS process are custom designed models and do not include transfer learning backbones such as VGG, ResNet, or DenseNet within the search space. In this study, transfer learning based models were evaluated separately as baseline comparators, whereas the NAS framework was used exclusively to discover task specific CNN architectures. Therefore, the transfer learning models and NAS generated CNN models represent two distinct experimental groups.

### 3.3 Transfer learning based CNN models

*ResNet‐50 (Residual Network)* is a deep learning architecture which uses residual blocks to address the issues of degradation and vanishing gradients that arise from the increased depth of training in deep neural networks. With a depth of 50 layers, this structure enables the more efficient and stable training of multi‐layered deep networks.^22^

*ResNet‐152's* architecture, consisting of 152 layers, enables networks to be trained to a depth that classical CNNs struggle to reach. The main innovation of this model is the ‘skip‐connection’ structures that are implemented every few layers. These structures prevent performance loss due to increasing depth by adding the input directly to the output of the next block, ensuring that the gradient flows smoothly between layers.^23^

*DenseNet‐121* is a CNN architecture that uses dense connections to improve gradient flow and increase parameter efficiency in deep learning models. This model offers better information flow and representational power by enabling each layer to reuse feature maps from all previous layers. DenseNet‐121 aims to achieve high accuracy by reducing information loss and gradient vanishing, both of which are particularly prevalent in deep networks.^24^

**FIGURE 5:**
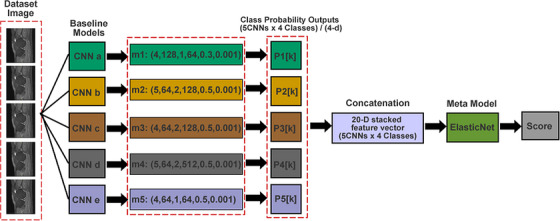
Stacking‐based late fusion architecture.

*VGG16* is a convolutional neural network (CNN) architecture consisting of 13 convolutional and three fully connected layers. The name is an abbreviation of ‘Visual Geometry Group 16’. Unlike previous models, which used filters of different sizes, VGG16 uses a 3×3 filter structure. This is considered its main advantage in preserving spatial resolution. Figure 5 illustrates the VGG16 architecture.^13^, ^25^, ^26^

*VGG19* is a deeper version of VGG16; while its architectural structure remains the same, it incorporates an additional 16 convolutional layers and 3 fully connected layers. By retaining the 3×3 convolutional filters, it enhances the ability to learn more intricate spatial features with additional layers, thereby augmenting the model's representational capacity while also escalating the computational expense.^27^, ^28^

### 3.4 Stacking strategy

Various studies have shown that, in most cases, ensemble methods can produce more successful predictions than basic models.^29^, ^30^, ^31^ In this study, a stacking‐based feature extraction process was employed to combine the strengths of multiple models and produce more reliable results. The stacking method comprises two layers. In the first stage, basic classifiers are trained. In the second stage, a higher‐level meta‐model processes these outputs to make the final decision.^32^, ^33^ The lower‐level models generate predictions based on the training data, which the meta‐model then uses to perform the final classification.

Figure 5 illustrates the structural workflow of the stacking‐based ensemble approach employed in this study. The process begins with input meniscus MRI images and proceeds through a multi‐classifier structure consisting of five NAS‐optimized base CNN models (CNN‐a–CNN‐e). Each CNN model has a unique set of hyperparameters (m1–m5), which are optimized using the NAS method. These hyperparameter configurations represent critical architectural and training‐related parameters, including the number of convolutional layers, filter sizes, dense layer units, dropout rates, and learning rates.

Each base CNN model processes the same MRI image independently and produces a four‐dimensional class probability vector corresponding to the four diagnostic categories. These outputs are denoted as P1(k), P2(k), P3(k), P4(k), and P5(k), where k represents the class index. Therefore, each CNN contributes four class probability values, and the outputs of the five CNN models are concatenated to form a 20‐dimensional stacked feature vector (5 models × 4 classes) for each sample. This feature vector is then transferred to the meta‐model layer.

ElasticNet is used as the meta‐learner in the proposed stacking framework. ElasticNet is a regularized linear model that combines L1 and L2 penalties and performs final classification by learning the contribution of each stacked prediction feature obtained from the base CNN models.^34^, ^35^ At this stage, the independent prediction patterns generated by the CNN models are evaluated jointly at a combined representation level. This enables the meta‐model to exploit model diversity and complementary class‐specific information.

The proposed approach employs a late fusion strategy, where the class probability outputs generated by multiple CNN models are combined and used as input to the ElasticNet meta‐model. Late fusion was preferred because it operates at the decision level and preserves the model‐specific decision patterns learned by independently trained CNNs. Unlike early fusion, which combines intermediate feature representations and may dilute architecture‐specific information, late fusion allows heterogeneous CNN architectures to be integrated more flexibly. In addition, compared with simple averaging or majority voting, stacking provides a learnable fusion mechanism that assigns different contributions to different base model outputs rather than treating all predictions equally.

The hyperparameters of the evaluated meta‐models were configured using random search, and model selection was based on validation performance. A diverse set of meta‐models was considered, including linear (ElasticNet), instance‐based (KNN), kernel‐based (SVM), tree‐based (RandomForest and XGBoost), and neural (MLP) approaches. Although advanced boosting methods such as LightGBM and CatBoost were not included, the selected models provide a representative comparison across different learning paradigms.

### 3.5 Proposed framework

This study uses an RS‐based NAS and stacking strategy. After the data processing phase, the RS algorithm is used to search all candidate CNN architectures. The five highest‐performing structures are then selected and combined in the stacking phase. During the NAS process, the RS algorithm (Algorithm 1) randomly samples hyperparameter combinations to generate different CNN configurations, which are then evaluated based on their validation performance. This prevents exponential growth in the hyperparameter search space and reduces computational cost. In each trial, the RS algorithm evaluates a different sequence of hyperparameters, storing the CNN configuration with the best validation performance. This ‘best’ structure is updated throughout the search.

ALGORITHM 1 **Random Search–Based Neural Architecture Search.**

**Table:** 

1:	archSearch(i, T, H, D):
2:	bestModel ← null
3:	bestAcc ← 0
4:	for t = 1 to T do
5:	h_t ← sampleRandom(H)
6:	M_t ← buildCNN(h_t)
7:	acc_t ← evaluate(M_t, D)
8:	if acc_t > bestAcc then
9:	bestAcc ← acc_t
10:	bestModel ← M_t
11:	end if
12:	end for
13:	return bestModel, bestAcc

During the stacking phase, the class probability vectors produced by the top five CNN models are concatenated to form a 20‐dimensional feature vector (5 models × 4 classes) for each MRI sample, which is then used as input to the ElasticNet meta‐learner. This model is used as a meta‐learner. ElasticNet then processes this additional information from the base models to produce the final classification score. Thus, the stacking structure combines the strengths of the five CNN models to achieve a more stable and accurate result. The pseudocode for this process is provided in the stacking() function in Algorithm 2.

ALGORITHM 2 **Stacking Procedure.**

**Table:** 

1:	stacking(S):
2:	F ← ∅
3:	M_meta ← Initialize ElasticNet Model
4:	for each sample k do
5:	f_k ← concatenate(p1[k], p2[k], p3[k], p4[k], p5[k]) ▹ 20‐D vector
6:	F.append(f_k)
7:	end for
8:	ŷ ← M_meta.fit(F)
9:	return ŷ

All deep learning models were implemented using the TensorFlow framework. The models were trained with the Adam optimizer (initial learning rate of 0.0001) and categorical cross‐entropy loss function, using a batch size of 32. Transfer learning models were trained for a maximum of 50 epochs, while for NAS‐generated CNN models, the effective training duration was determined by early stopping based on validation performance. Early stopping was applied with a patience of 10 epochs to prevent overfitting, and in most cases, the models converged before reaching the maximum number of epochs. To ensure reproducibility and reduce randomness effects, experiments were conducted within a repeated cross‐validation framework (10 runs × 5‐fold), where different random seeds were used in each run, and all data splits and model initializations were controlled accordingly.

### 3.6 Evaluation metrics

There are various criteria for the evaluation of performance metrics. These metrics include standard measurements such as accuracy, precision, recall, and F1 score.^36^ Table 3 shows the metrics used in the study.

**TABLE 3:** Performance metric formulas.

Metric	Formula	Explanation
Accuracy	$\frac{\left(TP+TN\right)}{(TP+TN+FP+FN}$	The total sample replacement rate of all correct predictions of the model.
Precision	$\frac{TP}{\left(TP+FP\right)}$	It shows how many of the positive predictions are actually positive.
Recall	$\frac{TP}{\left(TP+FN\right)}$	It shows how many true positives were predicted correctly.
F1‐Score	$\frac{2\;x\;(Precision\;x\;Recall)}{Precision+Recall}$	Harmonic mean of precision and recall.
MCC	$\frac{\left(TPx\;TN)-(FPx\;FN\right)}{\sqrt{\left(TP+FP)(TP+FN)(TN+FP)(TN+FN\right)}}$	It is a balanced performance metric that evaluates a classifier by considering all components of the confusion matrix.

Here:

TP (True Positive): True positive predictions

TN (True Negative): True negative predictions

FP (False Positive): False positive predictions

FN (False Negative): False negative predictions

These formulas contain commonly used metrics to evaluate the performance of the model.

## 4 RESULTS

### 4.1 Evaluation of five CNN models with transfer learning

In this study, a separate group of transfer learning based CNN models was evaluated as baseline comparators. These models were built on pre trained ImageNet architectures and fine tuned for the meniscus MRI classification task. The models were created using pre‐trained ImageNet base architectures, with fully connected structures added to their upper layers and optimised during training. Training was conducted within the TensorFlow deep learning framework on a system equipped with 128 GB of RAM and an AMD Ryzen 9 7950×3D processor. The dataset consists of four balanced classes, achieved using data augmentation methods. The 80/20 split was used only in preliminary experiments and is not part of the final evaluation protocol. All TL models were trained for a maximum of 50 epochs with a batch size of 32; however, thanks to the early stopping mechanism, the models generally converged after 10–25 epochs. The optimised hyperparameters included a learning rate of 0.0001 and a dropout rate of 40%. The accuracy (ACC), precision (PREC), sensitivity (REC), F1 score and Matthews correlation coefficient (MCC) were used to evaluate performance.

As shown in Table 4, the DenseNet121‐TL model achieved the strongest overall performance among the transfer learning based architectures. It provided the highest scores across nearly all evaluation metrics, including accuracy (86.25%), precision (0.8646), recall (0.8625), F1‐score (0.8627), and MCC (0.8172). In addition, it clearly outperformed the other models in terms of AUC (0.9832) and PR‐AUC (0.9602), indicating a more stable discrimination capability across different decision thresholds. The VGG16‐TL and VGG19‐TL models also delivered consistently high performance. With accuracy values of 86.00% and 84.25%, respectively, both architectures showed a balanced trade‐off between precision and recall. Their relatively strong AUC and PR‐AUC scores further suggest that VGG‐based models maintain reliable classification behavior when transfer learning is applied. In contrast, the ResNet50‐TL and ResNet152‐TL models yielded noticeably lower results. ResNet50‐TL reached an accuracy of 61.75%, while ResNet152‐TL achieved 62.00%, with comparatively weaker precision, F1‐score, and MCC values. Although their AUC scores were moderate, the overall metric distribution indicates that these models struggled to adapt effectively to the dataset under the same transfer learning setup. Overall, the results highlight that, within this experimental configuration, DenseNet and VGG‐based architectures are better suited for the task, whereas deeper ResNet variants do not provide a similar level of performance improvement.

**TABLE 4:** CNN models with transfer learning.

Model	ACC	PREC	REC	F1	MCC	AUC	PR‐AUC	Train Time (min)
VGG16‐TL	0.8600	0.8639	0.8600	0.8608	0.8140	0.9479	0.8914	11.3677
VGG19‐TL	0.8425	0.8528	0.8425	0.8445	0.7920	0.9535	0.9165	15.0348
ResNet50‐TL	0.6175	0.4872	0.6175	0.5356	0.5288	0.8073	0.5685	02.5994
DenseNet121‐TL	0.8625	0.8646	0.8625	0.8627	0.8172	0.9832	0.9602	09.9879
ResNet152‐TL	0.6200	0.6776	0.6200	0.6019	0.5121	0.8643	0.6696	04.7027

Table 4 presents the results of the transfer learning based baseline models, whereas Table 5 reports the performance of the top five custom CNN architectures generated through the Random Search based NAS process. These two tables therefore represent different experimental groups and are included to enable a clearer comparison between conventional transfer learning approaches and task specific NAS derived architectures.

**TABLE 5:** Performance of the best five architectures.

Model	Config	ACC	PREC	REC	F1	MCC	AUC	PR‐AUC	Train Time (min)
CNN‐a	(4,128,1,64,0.3,0.001)	0.9000	0.9009	0.9000	0.9003	0.8667	0.9848	0.9663	5.4909
CNN‐b	(5,64,2,128,0.5,0.001)	0.9150	0.9165	0.9150	0.9150	0.8871	0.9858	0.9693	2.0749
CNN‐c	(4,64,2,128,0.5,0.001)	0.9225	0.9259	0.9225	0.9220	0.8980	0.9832	0.9651	2.0786
CNN‐d	(5,64,2,512,0.5,0.001)	0.8975	0.9016	0.8975	0.8983	0.8642	0.9861	0.9682	2.1956
CNN‐e	(4,64,1,64,0.5,0.001)	0.8950	0.8955	0.8950	0.8939	0.8609	0.9854	0.9671	2.0548

*Note*: The complete performance results of all 94 candidate CNN architectures generated during the NAS process are provided in Table S1.

### 4.2 Performance evaluation of baseline models

This study used the RS algorithm for the hyperparameter search process to identify the most suitable architectures within the search space. The RS algorithm evaluated the performance of different architectural structures by randomly sampling from the defined parameter combinations. Table 5 presents the top five architectures and their accuracy, precision, sensitivity, F1 score and MCC values. According to Table 5, the CNN‐a model achieved the highest accuracy (0.9188) of all the models generated by RS. It also demonstrated balanced performance across all metrics, achieving a precision of 0.9191, a sensitivity of 0.9188, an F1 score of 0.9187 and an MCC value of 0.8918. The CNN‐b model, which ranked second, showed a similarly high success rate, with an ACC of 0.9031 and a PREC of 0.9066. The other models (CNN‐c, CNN‐d and CNN‐e) achieved accuracy values of 0.9000, 0.8938 and 0.8875 respectively. This demonstrates that the RS algorithm can produce consistent and competitive results using different parameter combinations.

The RS algorithm has been shown to efficiently scan a large search space and produce CNN architectures with high accuracy levels. The 0.9188 ACC achieved by the CNN‐a model in particular demonstrates the power and feasibility of RS as a hyperparameter optimisation strategy for this problem.

**FIGURE 6:**
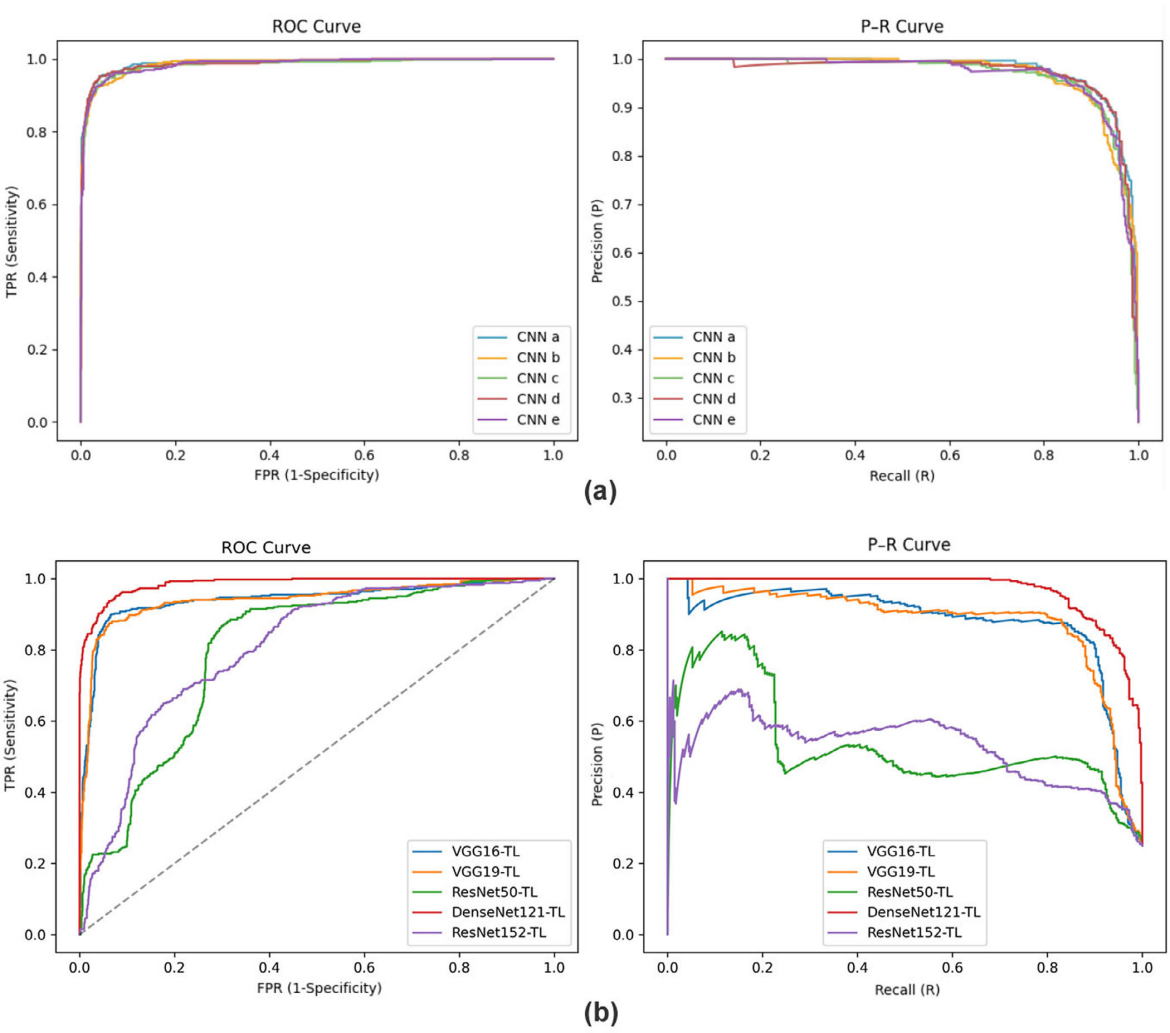
ROC and P‐R Curve of a) CNN models generated from NAS. b) CNN models with transfer learning.

Figure 6(a) shows the five custom CNN architectures generated by NAS. These exhibited very similar, high performance in the ROC and PR curves. The fact that almost all of the curves fall within an accuracy region above 0.9 indicates that these models provide robust and consistent classification performance. Similarly, the high precision‐recall levels of the PR curves reveal that the models can reliably recognise positive samples. This consistency confirms that the architecture space scanned by NAS is balanced, producing reliable models of similar quality. As can be seen in Figure 6(b), there are more pronounced performance differences among the TL models. In particular, DenseNet121‐TL clearly outperformed the others, achieving the highest level in both the ROC and PR curves. While VGG16‐TL and VGG19‐TL exhibited a stable and acceptable curve profile, ResNet50‐TL and ResNet152‐TL showed significant fluctuations, indicating inconsistencies in positive–negative sample differentiation. Overall, only the DenseNet121‐TL NAS models offer competitive performance, while the other transfer learning models lag behind the NAS architectures. This demonstrates that transfer learning does not guarantee success in every model, but can produce powerful results when the right structure is chosen.

The CNN models used in the experimental setup were trained on a system with 128 GB of RAM and an AMD Ryzen 9 7950×3D processor, running on the TensorFlow deep learning framework. This setup was used to accelerate training processes and support large‐volume experiments. This powerful hardware enabled the efficient execution of RS's structure, which requires a large number of variation trials. Furthermore, the hyperparameter search space used in the NAS process is summarised in Table 6. This includes a wide range of configurations, such as the number of convolution layers (ranging from 1 to 5), the number of filters per convolution layer (from 16 to 256), the number of fully coupled (dense) layers (1 or 2), the number of units per dense layer (from 64 to 512), the dropout rate (from 0.0 to 0.5) and the learning rate (selected from 1e–1, 1e–3 and 1e–4). This configuration enabled the RS algorithm to explore different architectural combinations and identify the best‐performing CNN models through a comprehensive search.

**TABLE 6:** Search space for the NAS.

Hyperparameter	Range
Number of Conv Layers	[1–5]
Filters per Conv Layer	{16, 32, 64, 128, 256}
Number of Dense Layers	{1, 2}
Dense Units per Layer	{64, 128, 256, 512}
Dropout	[0.0–0.5]
Learning Rate	{1e–1, 1e–3, 1e–4}

### 4.3 Establishment of a meta‐model using ElasticNet and performance of ensemble models

During the creation of the meta‐model, various machine learning algorithms were evaluated using 20‐dimensional stacked feature vectors derived from the class probability outputs of the five CNN models. Unlike simple aggregation methods (such as averaging or majority voting), the stacking approach we employed enables the strengths of different underlying models to be automatically combined. Consequently, the final meta‐model can deliver more stable, higher‐accuracy performance without human intervention. Table 7 shows the effect of different meta‐model candidates and illustrates how various ML algorithms behave as high‐level classifiers.

**TABLE 7:** Performance measurements on different algorithms (meta‐model).

MetaModel	ACC	PREC	REC	F1	MCC	AUC	PR‐AUC
KNN	0.9225	0.9256	0.9225	0.9229	0.8975	0.9768	0.9446
ElasticNet	0.9300	0.9328	0.9300	0.9304	0.9074	0.9913	0.9822
RandomForest	0.9150	0.9252	0.9150	0.9167	0.8891	0.9928	0.9816
XGBoost	0.9025	0.9070	0.9025	0.9027	0.8713	0.9890	0.9733
MLP	0.9000	0.9234	0.9000	0.9034	0.8730	0.9594	0.8922

As can be seen in Table 7, the ElasticNet meta‐model achieved the best overall classification performance among the evaluated meta‐models. It obtained the highest ACC, REC and F1 values, with an ACC of 0.9300, REC of 0.9300 and F1 score of 0.9304. In addition, ElasticNet produced the highest MCC value of 0.9074, indicating a more balanced and stable classification performance across classes. Although RandomForest achieved the highest AUC value of 0.9928, ElasticNet provided a slightly higher PR‐AUC value of 0.9822 and showed better overall performance in threshold‐dependent metrics. KNN also demonstrated competitive results, with an ACC of 0.9225 and F1 score of 0.9229, but remained slightly behind ElasticNet in terms of both MCC and PR‐AUC. XGBoost and MLP achieved lower ACC and F1 values compared with the other meta‐models, although their AUC values indicate acceptable discrimination capability. Overall, these results show that ElasticNet provides the most consistent and reliable performance for the proposed stacking framework in this experimental setup.

**FIGURE 7:**
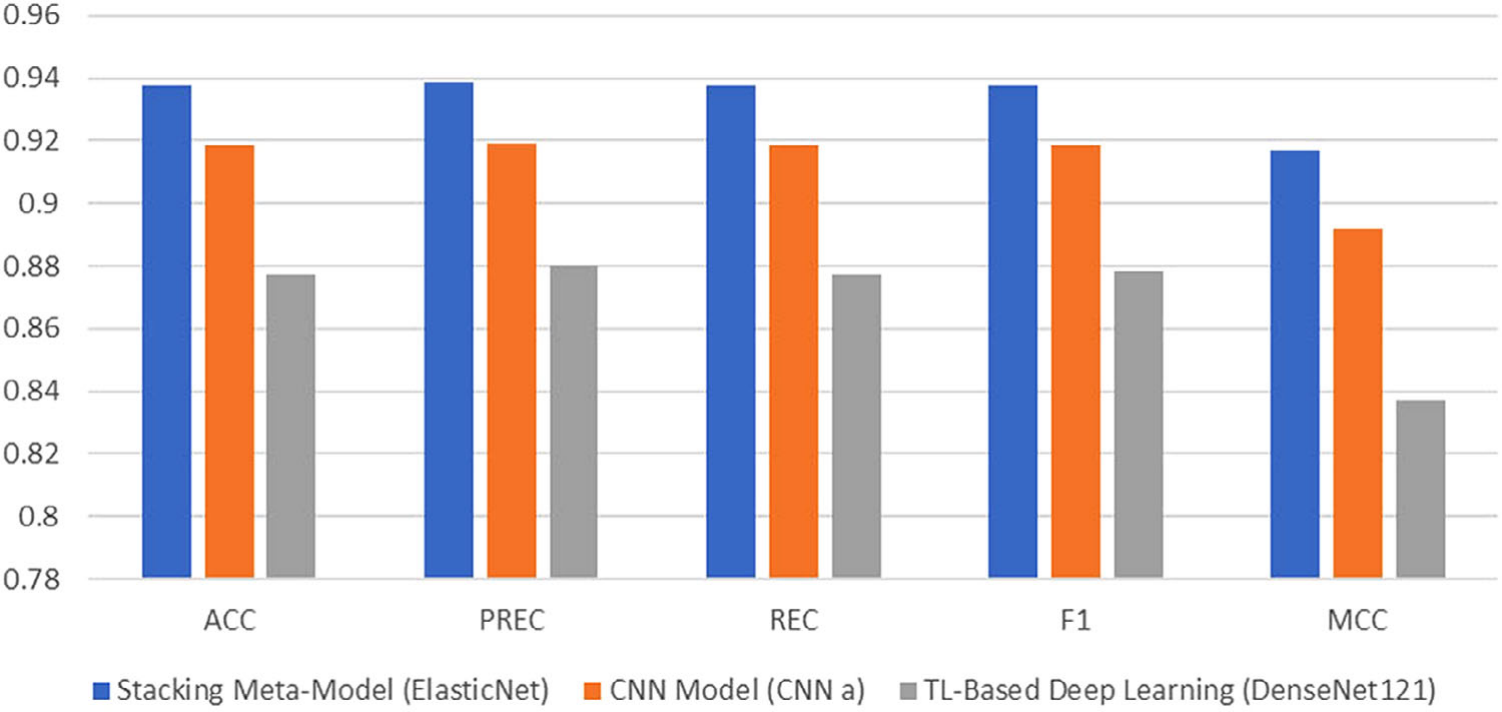
Best models across stacking, CNN, and deep learning approaches.

Figure 7 clearly illustrates the relationship between the three approaches. The stacking meta‐model (ElasticNet) achieves the highest scores in all metrics, demonstrating its superiority, particularly in MCC. This indicates that it is the model that best learns inter‐class balance. This suggests that the meta‐model can generate a more consistent and transferable representation by combining information from different base models. Despite being trained with a pure CNN architecture, the CNN model takes second place, offering strong performance in both ACC and F1 score. This suggests that the NAS‐derived structure fits the problem space well. DenseNet121, based on transfer learning, achieves lower scores than the other two models. The decrease in MCC in particular shows that the TL model is limited in its ability to learn the subtleties of data distribution.

### 4.4 Statistical evaluation and robustness analysis

To ensure a more reliable and unbiased evaluation of the proposed method, additional experiments were conducted using a repeated measures cross‐validation protocol. Specifically, a 5‐fold cross‐validation method was employed and 10 independent runs were performed with different random seed values. This experimental design aims to reduce dependence on a single data split and evaluate the model's stability and ability to generalise under different training conditions. Performance metrics such as accuracy (ACC), precision (PREC), sensitivity (REC), F1‐score, Matthews correlation coefficient (MCC), area under the ROC curve (AUC) and area under the precision‐recall curve (PR‐AUC) were calculated for each run and fold. The results were summarised using mean and standard deviation values across all runs. In addition to descriptive statistics, paired statistical tests were applied to assess the significance of differences in performance between models. In this context, paired *t*‐tests and non‐parametric Wilcoxon signed‐rank tests were used for metrics such as accuracy, F1‐score and MCC. The aim of these analyses is to determine whether the differences between the proposed stacking‐based model, the best NAS‐based CNN model and the transfer learning‐based model are statistically significant in terms of both metric values and overall performance.

Table 8 summarizes the overall performance of all models in terms of mean and standard deviation across 10 independent runs under a 5‐fold cross‐validation setting. The results clearly indicate that the proposed ElasticNet‐based stacking model achieves the highest performance across all evaluation metrics, including ACC, F1‐score, MCC, AUC, and PR‐AUC. In addition to achieving superior mean performance, the proposed model exhibits notably lower standard deviation values compared to baseline approaches, indicating a high level of stability and robustness across different runs. This consistency suggests that the stacking framework effectively mitigates variability inherent in individual models and produces more reliable predictions. While baseline models such as DenseNet121‐TL and the best NAS CNN achieve competitive results, their performance remains consistently below that of the proposed stacking approach. Furthermore, conventional ensemble strategies, including averaging and majority voting, show comparatively lower performance, highlighting the advantage of the learned fusion mechanism over simple aggregation methods. Overall, these findings demonstrate that the proposed stacking framework not only improves predictive performance but also enhances stability, making it a reliable and effective approach for the given classification task.

**TABLE 8:** Overall performance across 10 runs and 5‐fold cross‐validation.

Model	ACC (Mean ± Std)	F1 (Mean ± Std)	MCC (Mean ± Std)	AUC (Mean ± Std)	PR‐AUC (Mean ± Std)
Majority Voting Ensemble	0.7699 ± 0.0370	0.7485 ± 0.0514	0.7095 ± 0.0423	0.9206 ± 0.0091	0.8174 ± 0.0184
Averaging Ensemble	0.7939 ± 0.0236	0.7834 ± 0.0299	0.7368 ± 0.0273	0.9720 ± 0.0023	0.9345 ± 0.0053
DenseNet121‐TL	0.8366 ± 0.0072	0.8349 ± 0.0078	0.7869 ± 0.0090	0.9771 ± 0.0009	0.9494 ± 0.0020
Best NAS CNN	0.8302 ± 0.0223	0.8259 ± 0.0260	0.7797 ± 0.0271	0.9752 ± 0.0027	0.9417 ± 0.0067
ElasticNet‐Stacking‐Enhanced	0.9083 ± 0.0019	0.9084 ± 0.0018	0.8783 ± 0.0024	0.9883 ± 0.0008	0.9719 ± 0.0020

Table 9 presents the paired statistical comparisons obtained from the repeated validation protocol, which consisted of 10 independent runs with 5‐fold cross‐validation. The Wilcoxon signed‐rank test was used to compare ACC, F1‐score, and MCC values across models. The results demonstrate that the proposed ElasticNet‐based stacking model consistently and significantly outperforms all baseline approaches, including the best NAS CNN, DenseNet121‐TL, and conventional ensemble strategies. For all comparisons involving the stacking model, statistically significant improvements are observed (*p* < 0.05), with positive mean differences across all evaluated metrics. In particular, the stacking framework yields notable gains in MCC, indicating improved classification reliability and robustness across classes. Similarly, consistent improvements in ACC and F1‐score confirm that the performance gain is not limited to a single metric but is observed across multiple evaluation criteria. In contrast, the comparison between the best NAS model and DenseNet121‐TL does not show statistically significant differences for most metrics, suggesting that these baseline models exhibit comparable performance. This finding indicates that the observed improvement is primarily driven by the stacking mechanism rather than by the superiority of an individual base model. Overall, these results provide strong statistical evidence that the proposed stacking framework effectively leverages complementary information from base CNN models and produces more accurate and reliable predictions under a repeated 5‐fold cross‐validation setting.

**TABLE 9:** Paired statistical comparisons across 10 runs.

Metric	Comparison	Mean1	Mean2	Δ (Diff)	*p*‐value (Wilcoxon)
ACC	Stacking vs Best NAS	0.9083	0.8302	0.0781	0.0020
ACC	Stacking vs DenseNet	0.9083	0.8366	0.0717	0.0020
ACC	Stacking vs Averaging	0.9083	0.7939	0.1145	0.0020
ACC	Stacking vs Voting	0.9083	0.7699	0.1385	0.0020
ACC	NAS vs DenseNet	0.8302	0.8366	−0.0064	0.3750
F1	Stacking vs Best NAS	0.9084	0.8259	0.0825	0.0020
F1	Stacking vs DenseNet	0.9084	0.8349	0.0735	0.0020
F1	Stacking vs Averaging	0.9084	0.7834	0.1250	0.0020
F1	Stacking vs Voting	0.9084	0.7485	0.1599	0.0020
F1	NAS vs DenseNet	0.8259	0.8349	−0.0090	0.3223
MCC	Stacking vs Best NAS	0.8783	0.7797	0.0985	0.0020
MCC	Stacking vs DenseNet	0.8783	0.7869	0.0914	0.0020
MCC	Stacking vs Averaging	0.8783	0.7368	0.1415	0.0020
MCC	Stacking vs Voting	0.8783	0.7095	0.1688	0.0020
MCC	NAS vs DenseNet	0.7797	0.7869	−0.0072	0.4316

Table 10 presents the macro, micro, and weighted average performance metrics of the proposed model. The results indicate a consistently high level of performance across all evaluation measures. In particular, the close agreement between macro and weighted averages suggests that the model performs uniformly across all classes without exhibiting bias toward any specific category. This observation is further supported by the micro‐average results, which are highly consistent with the overall performance, indicating stable behavior at the sample level. Moreover, the high AUC and PR‐AUC values demonstrate that the model maintains strong discriminative capability and precision–recall balance across different classification thresholds. Overall, these findings confirm that the proposed approach achieves robust and balanced classification performance under varying evaluation perspectives.

**TABLE 10:** Macro, micro, and weighted average performance metrics of the proposed model.

Averaging Type	Precision	Recall	F1‐score	AUC	PR‐AUC
Macro	0.9092	0.9083	0.9084	0.9827	0.9596
Micro	0.9083	0.9083	0.9083	0.9843	0.9632
Weighted	0.9092	0.9083	0.9084	0.9827	0.9596

Table 11 presents an ablation‐style comparison of different modeling strategies, including individual CNN models, conventional ensemble methods, and the proposed ElasticNet‐based stacking framework. The results clearly demonstrate a progressive improvement in performance as the fusion strategy becomes more sophisticated. In particular, conventional ensemble approaches such as majority voting and averaging provide only limited performance gains, indicating that simple aggregation of model outputs is insufficient to fully exploit complementary information. In contrast, the proposed stacking model achieves a substantial improvement across all evaluation metrics, with the largest gains observed in MCC and PR‐AUC, which reflect improved class discrimination and precision–recall balance. Notably, the performance difference between individual CNN models (e.g., Best NAS and DenseNet121‐TL) remains relatively small, suggesting that the primary source of improvement does not stem from the choice of a single base model. Instead, the significant performance boost achieved by the stacking framework highlights the effectiveness of the learned fusion mechanism in capturing complementary strengths of multiple models. Overall, the ablation results provide strong evidence that the proposed stacking strategy is the key factor driving performance improvements, enabling more accurate, robust, and reliable predictions compared to both individual models and conventional ensemble techniques.

**TABLE 11:** Ablation‐style comparison of the proposed pipeline components and fusion strategies.

Component / Strategy	ACC	F1	MCC	AUC	PR‐AUC
Majority Voting Ensemble	0.7699	0.7485	0.7095	0.9206	0.8174
Averaging Ensemble	0.7939	0.7834	0.7368	0.9720	0.9345
Best NAS CNN	0.8302	0.8259	0.7797	0.9752	0.9417
DenseNet121‐TL	0.8366	0.8349	0.7869	0.9771	0.9494
Proposed ElasticNet‐Stacking	0.9083	0.9084	0.8783	0.9883	0.9719

**FIGURE 8:**
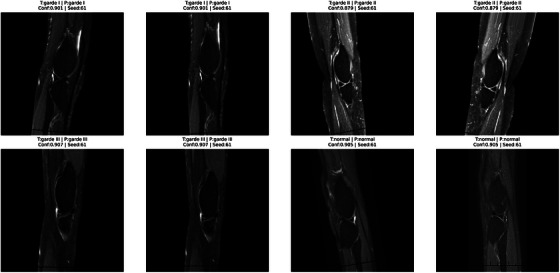
Representative correctly classified MRI samples from the test set across all classes.

Representative correctly classified MRI samples are presented in Figure 8. As shown, the model achieves high‐confidence predictions across all classes, including Grade I, Grade II, Grade III, and normal cases. The visual inspection of these samples indicates that the model is capable of capturing class‐specific structural patterns and signal characteristics within the meniscal region. In particular, the consistent agreement between true and predicted labels suggests that the learned feature representations are robust and discriminative across varying anatomical appearances.

**FIGURE 9:**
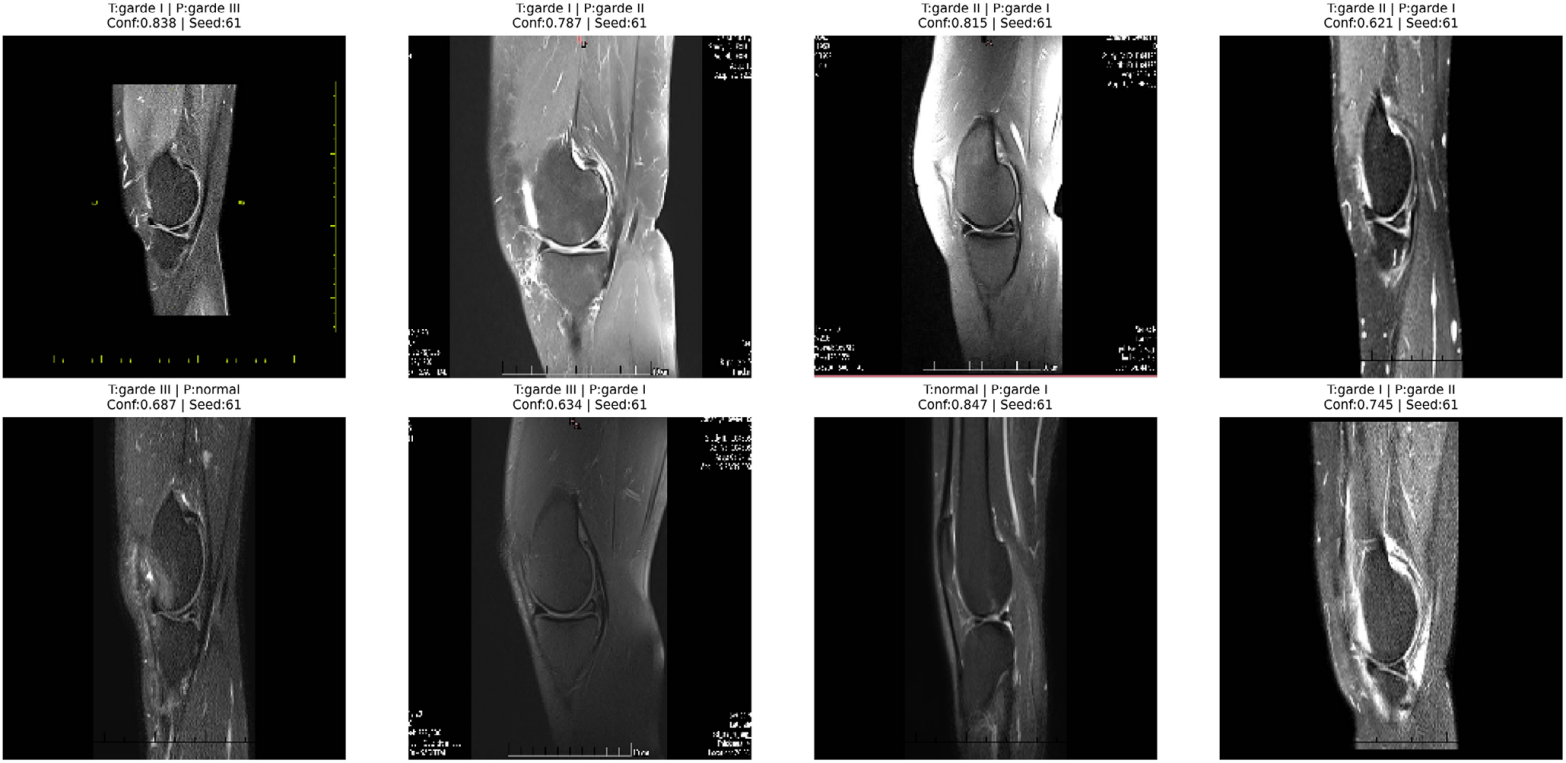
Representative misclassified MRI samples from the test set.

Representative misclassified MRI samples are shown in Figure 9. As observed, the model tends to confuse visually similar classes, particularly among Grade I, Grade II, and Grade III categories. This can be attributed to the gradual and subtle variations in meniscal signal characteristics, which may not always form clear class boundaries. In addition, a small number of normal cases are misclassified as pathological classes, suggesting that surrounding tissues or imaging artifacts may occasionally influence the model's predictions. These findings indicate that most errors arise from borderline cases rather than random misclassification. From a clinical perspective, such cases may also be challenging for visual interpretation because the signal alterations between adjacent grades can be subtle. Therefore, the misclassification pattern highlights the need for further validation using larger and more diverse datasets.

**FIGURE 10:**
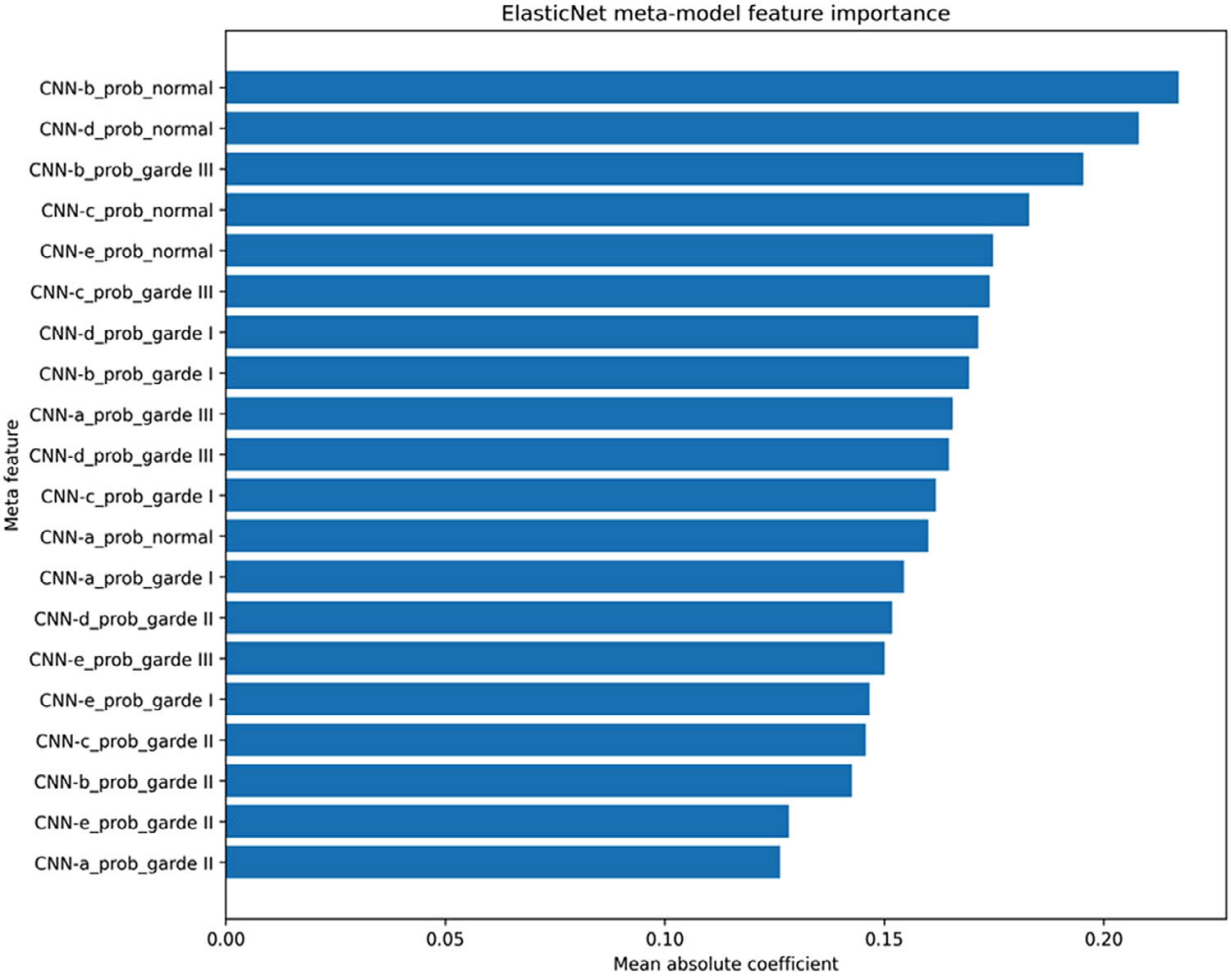
Feature importance of the ElasticNet meta‐model based on mean absolute coefficient values.

As shown in Figure 10, the feature importance results indicate that the stacking framework effectively prioritizes the most informative and reliable predictions generated by the base CNN models. In particular, outputs corresponding to clearly distinguishable classes, such as normal and Grade III, receive higher weights, whereas intermediate classes contribute less prominently. This behavior suggests that the meta‐model adaptively emphasizes more confident and discriminative features, thereby supporting overall classification robustness.

**FIGURE 11:**
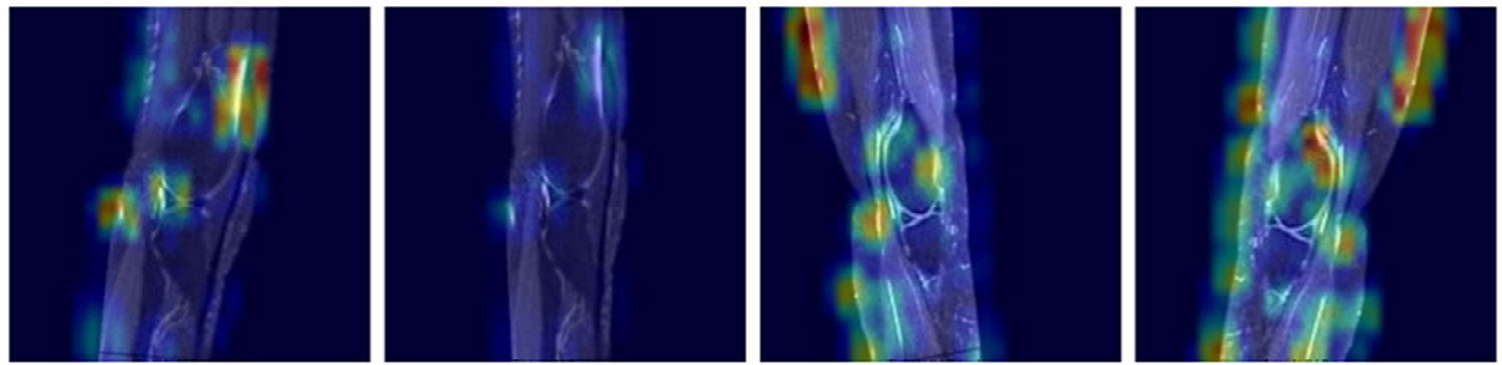
Representative Grad‐CAM visualizations for MRI samples.

As shown in Figure 11, the Grad‐CAM visualizations reveal that the CNN model consistently attends to the meniscal region and surrounding joint structures. This indicates that the model decisions are based on meaningful anatomical features, supporting the reliability of the proposed framework.

## 5 DISCUSSION AND CONCLUSION

A hybrid deep learning framework combining Random Search‐based Neural Architecture Search (RS‐NAS) and ElasticNet‐based stacking approaches has been proposed for classifying meniscus tears in MRI images into one of four categories. Results show that the proposed approach exhibits more stable and robust performance than single CNN models obtained with NAS or transfer‐based models.

In the single run evaluation, the ElasticNet‐based stacking meta‐model achieved ACC, F1‐score and AUC values of 0.9300, 0.9304 and 0.9913 respectively. This demonstrates that the class probabilities obtained from the selected CNN models can be effectively combined using the stacking structure. However, as results based on a single data split may be overly optimistic, the proposed model was also evaluated using 10 independent runs and a 5‐fold cross‐ validation protocol. The repeated validation achieved an ACC of 0.9083 ± 0.0019, an F1‐score of 0.9084 ± 0.0018, an MCC of 0.8783 ± 0.0024, an AUC of 0.9883 ± 0.0008 and a PR‐AUC of 0.9719 ± 0.0020. These results demonstrate that the proposed approach exhibits not only high peak performance, but also stable performance with relatively low variability under different data splits.

The difference between the single‐run evaluation and repeated cross‐validation results suggests that presenting a a single data split may provide an optimistic estimate. Therefore, the 10×5‐fold cross‐validation results more reliably reflect the model's realisation and generalisability. In particular, the low standard deviation values demonstrate that the proposed stacking structure is highly stable against different training/test splits. In clinical applications, the ability to generate results with different data conditions, as well as achieving the highest single‐run evaluation performance, is critical for clinical reliability.

Ablation results show that the performance improvement does not stem solely from a single robust CNN architecture, but primarily from the learnable late‐ fusion strategy. Classic ensemble approaches such as majority voting and averaging provide more limited performance; however, the ElasticNet‐based stacking model outperformed all other baseline approaches. This suggests that the stacking structure utilises more discriminative and reliable outputs more effectively than equally weighting the class probabilities from base CNN models.

Statistical analyses also support these findings. Wilcoxon signed‐rank test results show that the proposed ElasticNet‐Stacking model provides a significant advantage over the best NAS CNN and DenseNet121‐TL, the averaging ensemble and the majority voting methods in terms of the ACC, F1 and MCC metrics. However, the lack of a significant difference between the best NAS model and DenseNet121‐TL in most metrics suggests that the performance gain stems from the learnable combination mechanism of stacking rather than the superiority of a single model.

The study also presents explainability analyses. Grad‐CAM visualisations demonstrate that the CNN model primarily focuses on anatomically significant regions around the meniscus and joint when making decisions. Analysis of the feature importance of the ElasticNet meta‐model reveals that the stacking structure does not utilise all outputs from the base CNN models equally, but rather gives higher importance to class probabilities that are more discriminatory and reliable. These findings demonstrate that the proposed model produces high performance and that its decision‐making mechanism is interpretable to a certain extent.

However, the study has some limitations. The dataset was obtained from a single centre, which may introduce potential data bias depending on imaging protocols, device characteristics and patient population. To mitigate this, the dataset was carefully selected, images of low diagnostic value were excluded and data enhancement techniques were only applied to the training data. The model's robustness against different data splits was also tested using 10×5‐fold cross‐validation. Nevertheless, using single‐centre data may limit the model's generalisability to different clinical settings. Therefore, it is important to validate the model using multi‐centre, independent datasets with different devices and imaging protocols in future studies.

In conclusion, combining RS‐based NAS with an ElasticNet‐based stacking strategy offers a powerful, stable and interpretable approach to four‐class automated classification of meniscus MRI images. While high performance was achieved in the single‐centre evaluation, the results of repeated 5‐fold cross‐validation support the reliability and stability of the model. In this respect, the proposed system has the potential to reduce radiologists' workload and accelerate the diagnostic process in clinical decision‐making. In future, it is recommended that the model be validated using independent, multicentre datasets and tested in larger patient populations. Its integration into clinical workflows should also be evaluated.

## AUTHOR CONTRIBUTION

Ebubekir Seyyarer, Hasan Genç, and Faruk Ayata contributed equally to this study. All authors contributed to the conception and design of the study, data collection, analysis, interpretation of the results, and preparation of the manuscript. All authors reviewed and approved the final version of the manuscript.

## CONFLICT OF INTEREST STATEMENT

The authors declare that they have no known competing financial interests or personal relationships that could have appeared to influence the work reported in this study.

## DATA SOURCE AND ETHICS STATEMENT

This study used a dataset comprising 2,000 meniscus MRI images obtained at Fırat University Hospital. The data were collected from a clinical setting and are not derived from a publicly available repository.

All procedures involving human data were conducted in accordance with institutional and ethical standards. Ethical approval was obtained from the relevant ethics committee, and informed consent was secured in compliance with applicable regulations. All data were handled in a manner that ensures patient confidentiality and privacy, and no personally identifiable information was included in the study.

## Supporting information



Supporting Information

## Data Availability

The datasets used and/or analysed during the current study are available from the corresponding author on reasonable request.
