# Supplementary material for: A stacking based deep learning framework integrating random search neural architecture search for meniscus tear diagnosis
Source: J Appl Clin Med Phys. 2026 Jul 22;27(8):e70711. doi: 10.1002/acm2.70711 (PMC13389807; doi:10.1002/acm2.70711)
Supplement: Supplementary file 1 — Supporting Information [file ACM2-27-e70711-s001.docx]

**Table S1.** NAS-Generated CNN Architectures (All 94 Models)

| **Model** | **Conv Blocks** | **Filters** | **Dense Layers** | **Units** | **Dropout** |
| --- | --- | --- | --- | --- | --- |
| CNN 1 | 1 | 16 | 2 | 128 | 0.5 |
| CNN 2 | 4 | 16 | 1 | 128 | 0.3 |
| CNN 3 | 3 | 128 | 1 | 512 | 0.0 |
| CNN 4 | 1 | 64 | 1 | 512 | 0.0 |
| CNN 5 | 5 | 32 | 1 | 256 | 0.5 |
| CNN 6 | 4 | 64 | 2 | 256 | 0.0 |
| CNN 7 | 3 | 256 | 2 | 64 | 0.3 |
| CNN 8 | 5 | 128 | 2 | 64 | 0.3 |
| CNN 9 | 3 | 128 | 2 | 256 | 0.0 |
| CNN 10 | 3 | 32 | 1 | 128 | 0.0 |
| CNN 11 | 1 | 16 | 2 | 128 | 0.5 |
| CNN 12 | 4 | 16 | 1 | 128 | 0.3 |
| CNN 13 | 3 | 128 | 1 | 512 | 0.0 |
| CNN 14 | 1 | 64 | 1 | 512 | 0.0 |
| CNN 15 | 5 | 32 | 1 | 256 | 0.5 |
| CNN 16 | 4 | 64 | 2 | 256 | 0.0 |
| CNN 17 | 3 | 256 | 2 | 64 | 0.3 |
| CNN 18 | 5 | 128 | 2 | 64 | 0.3 |
| CNN 19 | 3 | 128 | 2 | 256 | 0.0 |
| CNN 20 | 3 | 32 | 1 | 128 | 0.0 |
| CNN 21 | 1 | 16 | 2 | 128 | 0.5 |
| CNN 22 | 4 | 16 | 1 | 128 | 0.3 |
| CNN 23 | 3 | 128 | 1 | 512 | 0.0 |
| CNN 24 | 1 | 64 | 1 | 512 | 0.0 |
| CNN 25 | 5 | 32 | 1 | 256 | 0.5 |
| CNN 26 | 4 | 64 | 2 | 256 | 0.0 |
| CNN 27 | 3 | 256 | 2 | 64 | 0.3 |
| CNN 28 | 5 | 128 | 2 | 64 | 0.3 |
| CNN 29 | 3 | 128 | 2 | 256 | 0.0 |
| CNN 30 | 3 | 32 | 1 | 128 | 0.0 |
| CNN 31 | 1 | 16 | 2 | 128 | 0.5 |
| CNN 32 | 4 | 16 | 1 | 128 | 0.3 |
| CNN 33 | 3 | 128 | 1 | 512 | 0.0 |
| CNN 34 | 1 | 64 | 1 | 512 | 0.0 |
| CNN 35 | 5 | 32 | 1 | 256 | 0.5 |
| CNN 36 | 4 | 64 | 2 | 256 | 0.0 |
| CNN 37 | 3 | 256 | 2 | 64 | 0.3 |
| CNN 38 | 5 | 128 | 2 | 64 | 0.3 |
| CNN 39 | 3 | 128 | 2 | 256 | 0.0 |
| CNN 40 | 3 | 32 | 1 | 128 | 0.0 |
| CNN 41 | 1 | 16 | 2 | 128 | 0.5 |
| CNN 42 | 4 | 16 | 1 | 128 | 0.3 |
| CNN 43 | 3 | 128 | 1 | 512 | 0.0 |
| CNN 44 | 1 | 64 | 1 | 512 | 0.0 |
| CNN 45 | 5 | 32 | 1 | 256 | 0.5 |
| CNN 46 | 4 | 64 | 2 | 256 | 0.0 |
| CNN 47 | 3 | 256 | 2 | 64 | 0.3 |
| CNN 48 | 5 | 128 | 2 | 64 | 0.3 |
| CNN 49 | 3 | 128 | 2 | 256 | 0.0 |
| CNN 50 | 3 | 32 | 1 | 128 | 0.0 |
| CNN 51 | 1 | 16 | 2 | 128 | 0.5 |
| CNN 52 | 4 | 16 | 1 | 128 | 0.3 |
| CNN 53 | 3 | 128 | 1 | 512 | 0.0 |
| CNN 54 | 1 | 64 | 1 | 512 | 0.0 |
| CNN 55 | 5 | 32 | 1 | 256 | 0.5 |
| CNN 56 | 4 | 64 | 2 | 256 | 0.0 |
| CNN 57 | 3 | 256 | 2 | 64 | 0.3 |
| CNN 58 | 5 | 128 | 2 | 64 | 0.3 |
| CNN 59 | 3 | 128 | 2 | 256 | 0.0 |
| CNN 60 | 3 | 32 | 1 | 128 | 0.0 |
| CNN 61 | 1 | 16 | 2 | 128 | 0.5 |
| CNN 62 | 4 | 16 | 1 | 128 | 0.3 |
| CNN 63 | 3 | 128 | 1 | 512 | 0.0 |
| CNN 64 | 1 | 64 | 1 | 512 | 0.0 |
| CNN 65 | 5 | 32 | 1 | 256 | 0.5 |
| CNN 66 | 4 | 64 | 2 | 256 | 0.0 |
| CNN 67 | 3 | 256 | 2 | 64 | 0.3 |
| CNN 68 | 5 | 128 | 2 | 64 | 0.3 |
| CNN 69 | 3 | 128 | 2 | 256 | 0.0 |
| CNN 70 | 3 | 32 | 1 | 128 | 0.0 |
| CNN 71 | 1 | 16 | 2 | 128 | 0.5 |
| CNN 72 | 4 | 16 | 1 | 128 | 0.3 |
| CNN 73 | 3 | 128 | 1 | 512 | 0.0 |
| CNN 74 | 1 | 64 | 1 | 512 | 0.0 |
| CNN 75 | 5 | 32 | 1 | 256 | 0.5 |
| CNN 76 | 4 | 64 | 2 | 256 | 0.0 |
| CNN 77 | 3 | 256 | 2 | 64 | 0.3 |
| CNN 78 | 5 | 128 | 2 | 64 | 0.3 |
| CNN 79 | 3 | 128 | 2 | 256 | 0.0 |
| CNN 80 | 3 | 32 | 1 | 128 | 0.0 |
| CNN 81 | 1 | 16 | 2 | 128 | 0.5 |
| CNN 82 | 4 | 16 | 1 | 128 | 0.3 |
| CNN 83 | 3 | 128 | 1 | 512 | 0.0 |
| CNN 84 | 1 | 64 | 1 | 512 | 0.0 |
| CNN 85 | 5 | 32 | 1 | 256 | 0.5 |
| CNN 86 | 4 | 64 | 2 | 256 | 0.0 |
| CNN 87 | 3 | 256 | 2 | 64 | 0.3 |
| CNN 88 | 5 | 128 | 2 | 64 | 0.3 |
| CNN 89 | 3 | 128 | 2 | 256 | 0.0 |
| CNN 90 | 3 | 32 | 1 | 128 | 0.0 |
| CNN 91 | 1 | 16 | 2 | 128 | 0.5 |
| CNN 92 | 4 | 16 | 1 | 128 | 0.3 |
| CNN 93 | 3 | 128 | 1 | 512 | 0.0 |
| CNN 94 | 1 | 64 | 1 | 512 | 0.0 |
